# Mechanisms of change of a multifaceted implementation strategy on fidelity to a guideline for the prevention of mental health problems at the workplace: a mechanism analysis within a cluster-randomized controlled trial

**DOI:** 10.1186/s13012-025-01437-4

**Published:** 2025-05-30

**Authors:** Andreas Rödlund, Anna Toropova, Rebecca Lengnick-Hall, Byron J. Powell, Liselotte Schäfer Elinder, Christina Björklund, Lydia Kwak

**Affiliations:** 1 Unit of Intervention and Implementation Research for worker health, Institute for Environmental Medicine, Karolinska Institutet, Stockholm, 171 77 Sweden; 2 Center for Mental Health Services Research, Brown School, Washington University in St. Louis, St. Louis, MO USA; 3 Center for Dissemination & Implementation, Institute for Public Health, Washington University in St. Louis, St. Louis, MO USA; 4 Division of Infectious Diseases, John T. Milliken Department of Medicine, School of Medicine, Washington University in St. Louis, St. Louis, MO USA; 5 Department of Global Public Health, Karolinska Institutet, Stockholm, 171 77 Sweden; 6 Centre for Epidemiology and Community Medicine, Region Stockholm, Stockholm, 104 31 Sweden

**Keywords:** Implementation science, Implementation strategy, Mental health, Fidelity, Implementation mechanisms, COM-B, Theoretical domains framework, Organizational intervention, Psychosocial risk management, Workplace intervention

## Abstract

**Background:**

Occupational guidelines exist to support workplaces with the prevention of mental health problems (MHP) among their staff. However, knowledge of effective implementation strategies to support their implementation is limited. This study experimentally tested whether a multifaceted implementation strategy – comprising an educational meeting, five workshops, implementation teams, small cyclical tests of change, and facilitation – improves fidelity to a guideline for preventing MHP in a school setting through the pathway of change of the Capability Opportunity Motivation-Behavior (COM-B)-model. To gain a more granular understanding of the mechanisms of change, the Theoretical Domains Framework (TDF) was used to specify mediators related to capability, opportunity, and motivation. This study tested whether the multifaceted strategy versus a discrete strategy (1) improves fidelity, (2) enhances capability, opportunity, and motivation over time, and (3) if the strategy’s effect on fidelity is mediated by capability, opportunity, and motivation.

**Methods:**

Fifty-five schools were randomly assigned to a multifaceted strategy or a discrete strategy. Fidelity was measured by questionnaires at baseline and 12 months, while capability, opportunity, and motivation were assessed three times within this period (directly after the educational meeting and at three and nine months). The Determinants of Implementation Behavior Questionnaire was used to assess TDF hypothesized mediators corresponding to the COM-B components. Separate pathways were analyzed for each mediator. Linear Mixed Modeling was employed to test the strategy’s effect on fidelity, and mediation analyses were conducted using the PROCESS Macro.

**Results:**

The multifaceted strategy led to improved fidelity at 12 months (B = 2.81, *p* < .001). Multifaceted schools reported higher scores for all mediators after nine months compared to schools receiving the discrete strategy. The effect of the multifaceted strategy on fidelity was partially mediated by all TDF mediators (*p* =  < .05) except for beliefs about consequences. Capability-related mediators, including skills (Proportion-mediated = 41%, *p* =  < .01) and behavioral regulation (Proportion-mediated = 35%, *p* =  < .001), accounted for the largest proportion of the effect, followed by the motivation-related mediator goals (Proportion-mediated = 34%, *p* =  < .01).

**Conclusions:**

The multifaceted strategy improved guideline fidelity by enhancing capability, opportunity, and motivation confirming the proposed function of COM-B. This study addresses calls for experimental evidence on how multifaceted implementation strategies achieve implementation outcomes.

**Trial registration:**

ClinicalTrials.org dr.nr 2020–01214.

**Supplementary Information:**

The online version contains supplementary material available at 10.1186/s13012-025-01437-4.

Contributions to the literature

- This study applied and experimentally tested a behavior change model to identify implementation mechanisms of a multifaceted implementation strategy aimed at improving fidelity to an evidence-based guideline for preventing work-related mental health problems in a school setting.
- Evidence is provided for multiple mechanisms of a multifaceted implementation strategy and for change in mediators over time.
- By assessing mediators through the COM-B and its matching Theoretical Domains Framework constructs and using an instrument with strong psychometric evidence, comparisons can be made across studies.

## Background

Mental health problems (MHP) are common in the working population and can contribute to work disability and early retirement [1]. The workplace is an important setting for the prevention of MHP, given that many MHP are caused by work-related risk factors such as high work demands, low control, and lack of support from management and colleagues [2, 3]. An evidence-based practice that can prevent work-related MHP is the systematic management of psychosocial risks in the work environment, which has been adopted in occupational health and safety legislation across countries worldwide [4, 5]. Due to the complexity of these legislations and the lack of concrete practical recommendations, occupational guidelines exist to support implementation [4, 5]. However, the use of these guidelines is low [6], and systematic reviews on occupational guidelines for the prevention of MHP show that knowledge of effective implementation strategies to support the implementation of guidelines is lacking [7, 8]. Consequently, the World Health Organization (WHO) has emphasized the need for more knowledge on how to implement these guidelines effectively [9].

A recent synthesis of systematic reviews on implementation strategies’ effectiveness in practice showed only small effects, and that the effect of the same strategy (e.g., training) often varies across studies [10]. To better understand why and how implementation strategies work, under what circumstances, and for whom, there is an increasing interest in understanding the mechanisms of change, i.e., *the processes or events through which an implementation strategy operates to affect desired implementation outcomes* [11, 12]. A mechanism links the strategy with the determinant it intends to address and explains how and why the strategy functions to achieve its effect [12]. Systematic reviews show that evidence of implementation strategies’ mechanisms is scarce [13, 14]. The majority of studies apply observational study designs, which hinders the establishment of a mechanism, and those studies that have applied experimental designs are unable to confirm their hypothesized mechanism [13, 14].

Since the publication of those reviews, research on implementation mechanisms has increased [15], and several studies have experimentally assessed implementation mechanisms. Williams and colleagues [16], for example, found that a theory-informed leadership implementation strategy improved implementation climate through leadership behavior, which in turn improved fidelity to the EBP [16]. Larson and colleagues [17] have evaluated a theory-informed, multifaceted motivational implementation strategy in a school setting and found that the strategy resulted in favorable effects in line with the Theory of Planned Behaviour and Health Action Process Approach, which in turn led to increased motivation to implement the EBP [17]. Recently published study protocols describe ongoing experimental studies to explore mechanisms of change and an Agency for Healthcare Research and Quality-funded conference series led to the development of a research agenda to advance the study of implementation mechanisms [18], further underscoring the growing interest in mechanisms-focused research in the field [19–21]. To further our understanding of effective implementation strategies for EBPs that prevent MHP in the workplace, the present study tested the mechanisms of change of a multifaceted implementation strategy to implement an occupational guideline for the prevention of MHP among school staff.

### Study context

The present study is part of a cluster-randomized controlled trial conducted in 55 schools in Sweden exploring the mechanisms of change of a theory-informed multifaceted implementation strategy used to support schools in their implementation of the *Guideline for the prevention of mental ill-health at the workplace* [22]. The guideline describes a systematic approach for the management of psychosocial risks in the work environment, which refers to a structured and ongoing process of identifying and assessing psychosocial risks in the work environment, developing and implementing risk reduction action plans, and evaluating and following up on these plans [23]. The multifaceted strategy includes an educational meeting, implementation teams, ongoing training through workshops, small cyclical tests of change, and implementation facilitation. The trial builds on the findings of our previous cluster-randomized controlled trial conducted among 19 schools in Sweden, which tested the effectiveness of the same multifaceted strategy but without implementation facilitation relative to a discrete strategy. The trial showed non-significant positive trends in improvements in fidelity to the guideline in favor of the multifaceted strategy [24]. Moreover, we saw that higher fidelity to the guideline was associated with lower levels of psychosocial work-environment risk factors, higher levels of psychosocial safety climate, and better health outcomes such as exhaustion [25]. Despite the overall positive trends observed in the first trial, the process evaluation conducted alongside the trial identified a lack of support from the school district as a barrier to the schools'implementation of the guideline. In this trial, implementation facilitation was added to the multifaceted implementation strategy to further enhance the school’s opportunity to implement the guideline [21], and to further understand the functioning of the implementation strategy, mechanisms of change were explored.

### Specifying hypothesized mechanisms of change

**Fig. 1:**
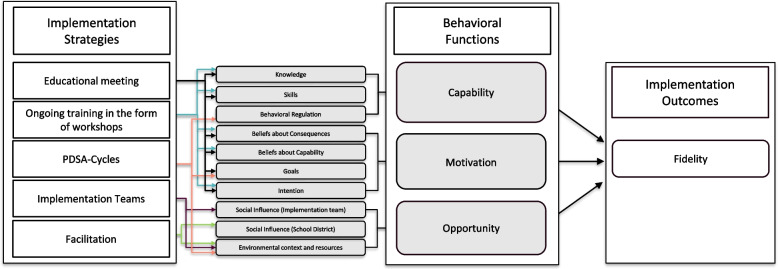
Figure 1 shows how the chosen implementation strategies are hypothesized to influence the TDF domains and the components of the COM-B model. *Note.* Hypothesis 1 (H1): The multifaceted strategy leads to larger improvements in guideline fidelity compared to the discrete strategy. Hypothesis 2 (H2): The multifaceted implementation strategy leads to larger improvements in capability, opportunity, and motivation compared to the discrete strategy. Hypothesis 3 (H3): The effect of the multifaceted implementation strategy on fidelity is mediated through capability (knowledge, skills, and behavioral regulation), opportunity (environmental context and resources, and social influence), and motivation (beliefs about consequences, beliefs about capability, goals, and intention)

The hypothesized mechanisms of change were specified in several steps. In the first step, prior to the first trial, barriers and enablers to the implementation of psychosocial risk management were identified in the literature and through planning workshops with school principals [26]. Identified barriers included a lack of knowledge of psychosocial work-environment risks and how to manage these risks [27, 28], unclear professional roles, lack of support from staff and school districts, and competing priorities [26]. A systematic approach to the management of psychosocial risks was identified as an enabler [26]. In the second step, the Capability Opportunity Motivation-Behaviour (COM-B) model was selected to inform the pathways of change (mechanisms of change). According to COM-B, behavior occurs in the presence of capability, opportunity, and motivation [29]. In accordance with the COM-B model, it was hypothesized that for schools to implement the *Guideline for the prevention of MHP at the workplace*, school management needs to have the capability, opportunity, and motivation to implement the guideline. In the third step, in a consensus process with experts, a multifaceted implementation strategy was developed to address school management’s capability, opportunity, and motivation to implement the guideline. The strategies were selected from and defined using the Expert Recommendations for Implementing Change (ERIC) compilation [30]. In the fourth step, which was part of the present trial, the original COM-B pathways identified were reassessed and refined based on the Theoretical Domains Framework (TDF) to provide a deeper understanding of the mechanism of change (see Fig. 1). While COM-B informs pathways of behavior change as a result of changing one or more of the COM-B components, the TDF provides a more granular understanding of the COM-B components by specifying domains for each of the components, namely capability (knowledge, skills, memory, attention and decision processes and behavioral regulation), opportunity (social influences, environmental context, and resources), and motivation (reinforcement, emotions, social/professional role and identity, beliefs about capabilities, beliefs about consequences, goals, and intentions) [31]. The domains of the TDF have previously been successfully mapped onto the COM-B, with excellent agreement [32]. As the TDF provides a more granular understanding of the COM-B components, it allows us to move beyond only identifying broad pathways of behavior change defined by the COM-B to identifying specific mechanisms of change that are needed to facilitate behavior change.

### Proposed mechanisms of change of the multifaceted implementation strategy

The multifaceted implementation strategy is comprised of strategies targeting each of the COM-B components and corresponding TDF domains as shown in Fig. 1. An educational meeting and ongoing training through workshops address capability and motivation to implement the guideline. The effectiveness of educational strategies on EBP has been summarized in several systematic reviews, including a Cochrane review [33]. Overall, educational meetings and workshops are assumed to improve professional practice with the underlying assumption that educational strategies improve knowledge and skills [33–35], as well as beliefs about consequences and beliefs about capability [33]. In this trial, the educational meeting and workshops are hypothesized to influence capability by providing information that increases knowledge on MHP and the guideline. This, in turn, enhances skills in how to prevent MHP in accordance with the guideline, which is expected to lead to positive beliefs about capability. Moreover, the educational strategies are hypothesized to influence motivation by providing information on the health consequences of working in accordance with the guideline, which is expected to lead to positive beliefs about consequences and the intention to implement the guideline. Finally, motivation is hypothesized to be influenced through goal formation specifying what is expected to be achieved when implementing the guideline [36].

To further facilitate behavior change, implementation teams will address opportunity by enabling social influence, and an environmental context and resources. Despite the lack of research examining the effectiveness of implementation teams on fidelity [37], positive effects of implementation teams on guideline implementation in clinical settings have been observed [38]. In line with an implementation team’s core functions identified in the literature [39], in the present study, the teams are expected to support the implementation of the guideline by adapting its recommendations to local contexts. Moreover, the implementation teams are hypothesized to enable an environmental context by creating an infrastructure of social support addressing principals’ perceived lack of support and unclear professional roles. This can occur, for example, through coordinating actions, ensuring that resources (e.g. time and staff) are in place, and clarifying who is responsible for implementation tasks. Moreover, implementation teams will engage in improvement cycles [40, 41].

Improvement cycles are operationalized through Plan-Do-Study-Act (PDSA) cycles and facilitate a structured approach to implementing the guideline. The PDSA cycles support principals with planning for change, addressing the principals’ perceived barrier to implementation, namely difficulty with executing plans [26]. Although evidence for PDSA cycles is limited due to low adherence in studies testing their effectiveness, studies have reported improved practice due to PDSA cycles [42]. PDSA cycles support the iterative development of small changes and quality improvements [43]. Research suggests that small-scale tests allow implementation teams to try actions and understand whether the applied changes work [43], which has shown to increase the team’s confidence that the implemented action(s) leads to improvements [43, 44]. This approach, when used as intended, promotes a structured approach to implementing change by providing, among others, role clarity for the implementation (such as who, what, where, and when) [45] and by identifying necessary refinements for the next cycle [44]. In this trial, the formation of PDSA cycles is hypothesized to influence capability, opportunity, and motivation. Conducting PDSA cycles is expected to influence capability through behavioral regulation by providing a structure for action planning, problem-solving, goal-setting and self-monitoring leading to habit formation. Motivation will be influenced through goal formation specifying what is expected to be achieved through the PDSA cycles [36]. Finally, PDSA cycles are hypothesized to influence opportunity by enabling an environmental context by providing a structured approach specifying how, where, and by who the guideline will be implemented.

In the present trial, implementation facilitation was added to the multifaceted implementation strategy to address the lack of support that the schools experienced in our previous study [22]. At each municipality, an internal facilitator will support the implementation teams [22]. A systematic review and meta-analysis showed that facilitation is an effective strategy for implementing EBPs and that it is intended to support implementation by tailoring actions to specific needs and contexts [46]. Moreover, a recent scoping review on implementation facilitation reported that support and problem-solving (e.g., identifying barriers and solutions to overcome them) are core functions of facilitation during the implementation phase [47]. In this trial, the internal facilitator was hypothesized to influence opportunity through social influence, including the enablement of social support and organizational commitment from the school district, but also by enabling supervision and feedback from the school district. The internal facilitator was further hypothesized to influence opportunity by enabling a context within which knowledge is exchanged, barriers to implementation are identified, and processes or solutions to overcome those barriers are developed, applied, and refined.

### Study aim and hypotheses

This trial aimed to explore the implementation mechanisms through which a discrete versus a multifaceted implementation strategy operate to affect fidelity to the *Guideline for the prevention of mental ill-health at the workplace* [22]. The overall aim was to explore whether the effects of the implementation strategies on fidelity are mediated by capability, opportunity, and motivation. In the effectiveness study on fidelity to the guideline recommendations, we found that the multifaceted strategy was more effective than the discrete strategy in improving fidelity to nearly all the guideline indicators (Rödlund et al., under review). In the present study, we tested whether the effect of the multifaceted strategy on guideline fidelity was mediated by capability, opportunity, and motivation.

The first hypothesis was that the multifaceted implementation strategy compared to the discrete strategy would lead to larger improvements in fidelity between baseline and 12 months (H1). The second hypothesis was that the multifaceted implementation strategy would lead to larger improvements in capability, opportunity, and motivation over time compared to the discrete strategy (H2). The third hypothesis was that the effect of the multifaceted implementation strategy on fidelity would be mediated by capability, opportunity, and motivation (H3).

## Method

This study is part of a waiting list cluster-randomized controlled trial with before and after measurements [22]. The trial enrolled 55 schools from four municipalities. The schools were randomized (1:1 ratio) to receive either the multifaceted (ARM 1) or the discrete implementation strategy (ARM 2) in year 1. ARM 1 received all strategies during year 1 (2021–2022), while ARM 2 formed implementation teams and participated in the educational meeting in year 1 and received the complete bundle of implementation strategies (workshops, improvements cycles and internal facilitator) during year 2 (2022–2023) [22]. The current study reports on year 1. The study was funded by the Swedish Research Council for Health, Working Life and Welfare (2021–01828) and approved by the Swedish Ethical Review Agency (2021–01828). Trial registration: NCT05019937, Unique Protocol ID: 2020–01214. Registered 9 August 2021, https://register.clinicaltrials.gov/. Appendix 1 shows a CONSORT checklist for reporting randomized trials.

### Procedure

The primary implementation outcome, fidelity to the guideline, was assessed at the school staff level at two time-points, baseline (T_0_) and the 12-month follow-up (T_4_), through a web-based survey. Staff participants were employed by the school, including, among others, teachers, administrators, paraprofessionals (e.g., teacher aides, teaching assistants), and special education assistants. Individuals were excluded if they were not directly employed by the school, such as cleaning and kitchen personnel.

**Fig. 2:**
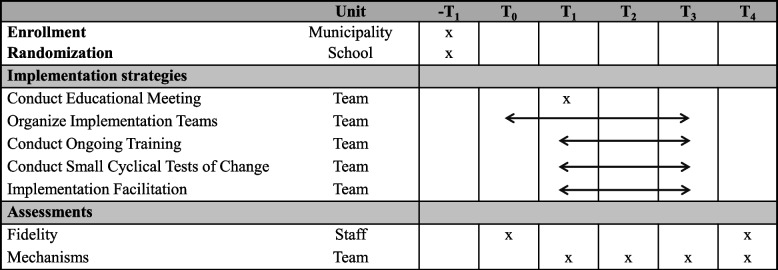
Study timeline

To assess the effects of the implementation strategies on capability, opportunity, and motivation to implement the guideline and on the mechanisms of change, participants belonging to the discrete or multifaceted strategy who participated in the educational meeting completed a survey at four time points. Figure 2 shows the study timeline. Surveys were distributed on-site at time points corresponding to the end of the education meeting (T_1_; October–November 2021), digitally at the end of workshop 3 (T_2_ at 3 months; January–February 2022), on-site (multifaceted group) and digitally (discrete group) at the end of workshop 5 (T_3_ at 9 months; May 2022), and digitally 12 months after baseline (T_4_; September 2022). Each survey was timed based on theory-driven expectations of hypothesized mechanisms. As a result, those hypothesized mediators (*n* = 4) that were not addressed by the strategies at T_1_ were only assessed at T_2_ to T_4_. The survey distributed at T_1_ assessed the hypothesized mediators targeted by the educational meeting, namely those TDF domains related to the COM-B components capability (knowledge and skills) and motivation (beliefs about the capability, beliefs about consequences, intention, and goals). The survey distributed at T_2_ aimed to assess the above-mentioned TDF domains, which were expected to change as a result of workshops 1–3. In addition, the survey assessed the hypothesized mediator behavioral regulation targeted by the PDSA and those hypothesized mediators related to opportunity (social influence implementation team, social influence school district, and environmental context and resources) that were mainly targeted by the implementation team and internal facilitator. The survey at T_3_ (workshop 5) was conducted after participants had been exposed to all implementation strategies, at which point it was expected that all hypothesized mediators would be fully activated. Since we anticipated that all implementation strategies were necessary to improve all mechanisms, T_3_ was selected as the time point relevant for testing the mechanisms in the present study. A more detailed description of the procedure is found in the study protocol [22].

### Interventions

#### The guideline for the prevention of mental ill-health at the workplace

The guideline being implemented is a Swedish occupational health guideline developed in a collaboration between researchers, employer representatives, and occupational health practitioners and is based on the best available practice and research evidence [23]. The guideline consists of three recommendations for the prevention of work-related MHP [23]. These recommendations are: (1) workplaces should have well-established policies for psychosocial risk management, (2) managers should have knowledge about the relationship between psychosocial work-environment risk factors and MHP, and (3) workplaces should regularly assess their psychosocial work environment and intervene on identified risk factors. A more detailed description of the guideline recommendations and related target activities can be found elsewhere [22, 26].

#### Implementation strategies

##### Organize implementation teams 48– ERIC cluster Develop stakeholder inter-relationships [30, 48]

Implementation teams were defined as a local team of school management and staff who are responsible for implementing the guideline within their school. Prior to the educational meeting (August to September 2021), the principals in the multifaceted group formed an implementation team within their schools. The principal received instructions by email to specify the team members'names, their roles at the school, and a motivation for their inclusion in the implementation team. The team consisted of 4–5 people representing a mix of staff, for example, principals, assistant principals, teacher union representatives, and health and safety officers. The teams were instructed to participate in the educational meeting, the workshops, and the PDSA-cycles. Moreover, they were encouraged to have regular meetings during the study period, yet no instructions were given by the research team on the length and/or frequency of these meetings. The principal of the schools belonging to the discrete group received the instructions (August to September 2021) to form a team of 4–5 people to participate in the educational meeting in year 1.

##### Conduct an educational meeting 15– ERIC cluster Train and educate stakeholders [30, 48]

Conducting an educational meeting was defined as providing information on mental ill-health, the guideline, and the advantages of adhering to the guideline for the prevention of mental ill-health. The implementation teams participated in a half-day educational meeting, which was led by an implementation researcher (LK) and an occupational health researcher (CB) and was held at each participating municipality in October 2021. The meeting included education on MHP, the guideline recommendations, and the benefits of working in accordance with the guideline. It also involved practical activities such as group discussions and exercises (e.g., making an implementation plan and formulating Specific, Measurable, Achievable, Relevant, and Time-bound (SMART) goals). At the end of the meeting, a lecture was given on what is needed to succeed with the developed plans, and potential implementation barriers and facilitators were introduced.

##### Conduct ongoing training in the form of workshops 19– ERIC cluster Train and educate stakeholders [30, 48]

Conducting ongoing training in the form of workshops was defined at conducting training in the guideline and PDSA cycles in an ongoing way. The research team organized five workshops at each participating municipality, each 150 min, between October 2021 and June 2022. The first workshop was held two weeks after the educational meeting. Each workshop focused on a specific guideline recommendation, and the last workshop focused on providing the implementation teams with a structure to continue working in accordance with the guideline. The workshops included providing ongoing training on how to prevent work-related MHP in accordance with the guideline, and how to implement the guideline recommendations by using PDSA cycles. The workshops combined lectures, discussions, and practical exercises and were divided into three modules. The first module of workshops 2–5 involved the implementation teams presenting their progress in line with their PDSA cycles. The second module focused on the content of the guideline and its recommendations, including a combination of lectures and exercises. The last module provided further knowledge and skills for implementing the guideline through exercises on setting goals and developing implementation plans in accordance with PDSA. Due to the COVID-19 pandemic, workshops 3 and 4 were conducted digitally via Microsoft Teams [49]. Moreover, in one municipality, two workshops were given simultaneously in a slightly shortened version without compromising the content of the workshops. This was a deviation from the study protocol [22].

##### Conduct cyclical small tests of change 14– ERIC cluster Use evaluative and iterative strategies [30, 48]

Conducting cyclical small tests of change was operationalized as PDSA cycles, defined as a method for structuring iterative development of change [43]. The first PDSA cycle started during the first workshop in October 2021. Following the PDSA structure [43, 44], the implementation teams compared their current routines for the prevention of MHP with the guideline recommendations, specified a goal for changing their management of psychosocial work environment risks to be more in line with the guideline recommendations, identified barriers and facilitators to attain the goal, identified what changes are needed to attain the goal and developed a plan that describes the actions that are needed and who is responsible for conducting the actions. Between the workshops, the implementation teams implemented their plans and followed their progress. At the following workshop, the teams studied whether their plans worked as planned or if any adjustments were necessary. To support the teams with their PDSAs, each team received a PDSA planning template during each workshop. Between the workshops, the implementation teams were reminded by the research team to complete their PDSA templates.

##### Implementation facilitation 10 – ERIC cluster Provide interactive assistance [30, 48]

Internal facilitation was defined as a process of enabling implementation teams in their efforts to implement the guideline into practice [50]. At the start of the trial, each municipality selected one or two representatives from the municipality to act as internal facilitators, for example, the head of education, human resource specialists, and school-district principals. Important competencies of the internal facilitator included communication skills (e.g., interacting with the implementation teams and asking questions to understand the teams’ needs and concerns) and competencies related to building relationships and creating a supportive environment (e.g., motivating), and building structures and processes that support implementation (e.g., problem identification and solving skills). Overall, the task of the facilitator was to support the implementation teams with implementing the guideline. Activities of the facilitator included praising teams for their implementation progress and encouraging them to assess their own efforts, share their successes, solve problems, and create their own strategies. In addition to supporting teams with identifying and addressing problems, e.g., by providing implementation resources and practical support, for example, by providing information about existing tools such as documents and checklists developed by the municipality. Facilitators also participated in the educational meeting and workshops and were instructed to schedule meetings with implementation teams based on their needs.

### Measures

#### Descriptive variables

Descriptive variables assessed by a survey at baseline were age, gender, number of years working at the school, and number of years working within the profession. Self-reported stress was assessed with a single item with 5-point response anchors ranging from (1) “not at all” to (5) “very much” Self-perceived health was assessed with a single question from the SF-12 Health Survey: “In general, would you say that your health is…” with response options ranging from (1) “excellent” to (5) “poor” [51].

#### Fidelity to the guideline

Fidelity to the guideline was assessed by a web survey of all school staff, which included ten statements on the schools' fidelity to the guideline recommendations (e.g., the school has carried out efforts for employees to recognize the content of work environment documents" and "school/work management has sufficient competence in the area of psychosocial risks"). School staff reported on a 5-point Likert scale ranging from 1 (strongly disagree) to 5 (strongly agree) whether they agreed with the statement. The statements assessing fidelity were constructed by the research team, and pilot tested in the first trial [24, 26]. The survey underwent further refinement after interviews with stakeholders [22]. Refinements to the survey focused on two areas. First, the survey instructions were revised to include explanations of terms such as work environment document, and risk assessment, which participants had found unclear. Second, the language was made more precise by replacing vague references like “we” and “others” with specific prompts that distinguish between different roles and stages in work environment management, for example leadership and employees. Fidelity to guideline Recommendation 1 was assessed with three statements (Cronbach alpha = 0.92), fidelity to Recommendation 2 was assessed with a single statement, and Recommendation 3 was assessed with six statements (Cronbach alpha = 0.91). The answers to all statements were summed (Cronbach alpha = 0.91) to generate a total fidelity sum score ranging from 5 to 50. More information about the survey can be found in previous publications [22, 24, 26].

#### Mechanisms of change

The Determinants of Implementation Behavior Questionnaire (DIBQ) [52] was developed to assess the behavioral determinants of the 12-domain version of the Theoretical Domains Framework (TDF). Previous studies have mapped TDF domains to COM-B components and the TDF has been used in prior research to explain the latent components of COM-B [32, 53]. The DIBQ has been tested in previous studies and has shown good validity and reliability [52, 54].

In the present study, an adapted version of DIBQ was used to assess 9 of the 12 domains of TDF. These domains corresponded to the COM-B components capability (3 domains), opportunity (2 domains) and motivation (4 domains). Consistent with previous research, capability was assessed through the TDF domains: knowledge (3 statements), skills (3 statements), and behavioral regulation (3 statements). Opportunity was assessed through the TDF domains: social influence, environmental context and resources (3 statements). Social influence was assessed from two perspectives; whether respondents perceived that they received support from the implementation team (3 statements) and whether they received support from the school district (3 statements). Motivation was assessed with the TDF domains: beliefs about capabilities (3 statements), beliefs about consequences (3 statements), intentions (3 statements), and goals (3 statements). Response to each statement was given on a 7-point scale ranging from "strongly disagree" to "strongly agree" [52, 54]. Cronbach alpha values for each TDF domain ranged from 0.77 to 0.95, indicating acceptable internal reliability [55].

### Data analysis

To confirm the effect of the multifaceted strategy found in our previous study (Rödlund et al., under review) on fidelity sum score on both staff level and school levels, Linear Mixed Modeling (LMM) was used. Group and time variables represented fixed factors, and group*time interaction indicated the intervention effect.

**Fig. 3:**
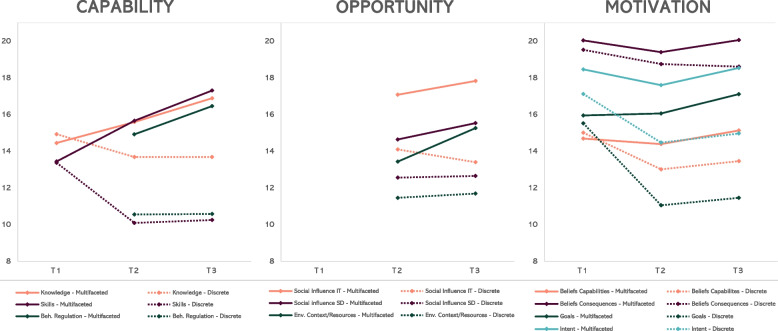
The effect of the implementation strategies on the nine TDF domains matching the COM-B model. Note: Mean values are reported on the y-axis, and the time point is on the x-axis. Social influence was assessed from two perspectives; whether respondents perceived that they received support from the implementation team (IT) and whether they received support from the school district (SD)

The development of the hypothesized mediators over time was represented by graphs (Fig. 3). Mediation analysis estimating the indirect effect of the strategy on fidelity through the hypothesized mediators was conducted using PROCESS Macro for SPSS by Andrew Hayes [56]. For this analysis, aggregated school-level outcome data (fidelity) was used. Each of the nine TDF hypothesized mediators was analyzed separately in linear regression models (see Table 3). Direct and total effects were also estimated. The proportion-mediated statistics were calculated by dividing the indirect effect by the total effect. The PROCESS macro handled missing data using listwise deletion, meaning that if a participant has missing data on any variable included in the model, they were excluded from the analysis [56]. Statistical power and sample size calculation for the mediation analysis is reported elsewhere [22].

## Results

### H1: The effectiveness of strategies on fidelity

A total of 2276 participants completed the baseline survey on fidelity (multifaceted group: 53%, discrete group: 47%, average response rate 83.8%). 1891 participants completed the survey on fidelity at T_4_ (participation rate = 76.8%, including 265 new participants). 1626 participants completed the survey at both time points (response rate = 85.9%)(Table 1).

**Table 1:** Sample characteristics at baseline for school staff and T_1_ for participants attending the educational meeting

Characteristics	Multifaceted Group	Discrete Group
**School Staff**	1218	1058
Age (mean)	48.4	48.7
Gender		
Female	70.7 %	71.0 %
Professional Title (n)		
Teacher	736	660
Other school staff	464	381
Work experience, years (mean)		
Within the occupation	15.2	15.1
Within the school	7.7	8.2
General Health (1–5; mean)	2.7	2.7
Stress (1–5; mean)	3.0	3.1
**Educational Meeting Participants**	104	103
Age (mean)	50.3	50.0
Gender		
Female	74 %	78 %
Professional Title (n)		
Principals (inc. assistant principals)	30	30
Other management (e.g., administrative manager)	1	5
Line manager	8	15
Health & Safety Officers	20	16
Union representatives	14	9
Teachers	21	21
Other school staff (e.g., support staff)	10	7
Work experience, years (mean)		
Within the occupation	20.2	16.2
Within the school	10.6	10.1
General Health (1–5; mean)	2.6	2.5
Stress (1–5; mean)	3.0	3.2

Anchors for General Health range from 1 = Excellent to 5 = Poor, and anchors for Stress range from 1 = Not at all to 5 = Very much

Table 2 depicts the mean fidelity scores per group over time and the impact of the strategies on fidelity over time. Larger and statistically significant improvements in fidelity were observed for the multifaceted group than for the discrete group (Staff level: B = 2.23, CI = 1.14 to 3.51, *p* < 0.001, School level: B = 2.81, CI = 2.49 to 3.12, *p* < 0.001).

**Table 2:** Fidelity scores per group over time and the comparative effectiveness between the multifaceted versus the discrete implementation strategy over time

		T_0_ (*N*= 2276)		T_4_ (*N*= 1891)				
		Mean	SD	Mean	SD	B	95% CI	*p*
Fidelity – staff level	Multifaceted group	19.8	11.4	25.2	12.6	2.23	1.14 to 3.51	<.001
	Discrete group	19.4	12.1	22.0	11.9			
Fidelity – school level	Multifaceted group	19.8	3.2	25.1	4.0	2.81	2.49 to 3.12	<.001
	Discrete group	19.4	3.9	21.8	4.4			

Coefficient = unstandardized Beta, CI = 95 % Confidence Interval

### H2: The multifaceted strategy’s effect on mediators over time

At T_1_, all 207 participants (multifaceted group: 52%, discrete group 48%) taking part in the educational meeting completed the survey on mediators, 191 participants completed the survey at T_2_ (comprising 13 new participants belonging to the multifaceted group), and 179 participants completed the survey at T_3_. Figure 3 illustrates the change in TDF domains per group over time, with mean values reported on the y-axis. For the TDF domains related to capability, the multifaceted group improved in all three domains, resulting in higher mean values at T_3_ than those observed in the discrete group. The discrete group decreased in all three capability-related domains compared to T_1_. For the TDF domains related to opportunity, the multifaceted group improved in all domains between T_2_ and T_3_, with higher reported means in all domains compared to the discrete group. The discrete group improved in two TDF domains of opportunity. For the TDF domains related to motivation, the multifaceted group improved in all four domains, resulting in higher mean values at T_3_ than those observed in the discrete group. The discrete group reported lower mean values at T_3_ than at T_1_ for all TDF domains related to motivation.

Table 3 shows the effect of the implementation strategies (predictor) at baseline on the TDF-domain (mediator) at T_3,_ the effect of the mediator on fidelity (outcome) at T_4_, and the mediation effect. The largest effect of the multifaceted strategy was observed on capability-related domains skills (B = 7.13, CI 6.09 to 8.17) and behavioral regulation (B = 5.99, CI 4.87 to 7.12). The largest effect of the multifaceted strategy on opportunity-related domains was observed for social influence from the implementation team (B = 4.51, CI 3.50 to 5.52) and for motivation-related domains the largest effect was observed for goals (B = 5.83 CI 4.71 to 6.95).

**Table 3:** Results of the mediation analysis for the nine TDF domains comprising the COM-B model

Mediator	Predictor-mediator effect (95 % CI)	Mediator-outcome effect (95 % CI)	Average indirect effect (95 % CI)	Average direct effect (95 % CI)	Average total effect (95 % CI)	Proportion mediated (%)
Capability						
Knowledge	3.22 (2.21–4.22)	0.25 (0.06–0.44)	0.81 (0.25–1.43)	3.94 (2.52–5.36)	4.75 (3.45–6.06)	17 %
Skills	7.13 (6.09–8.17)	0.28 (0.09–0.46)	2.01 (0.73–3.37)	2.78 (0.96–4.61)	4.80 (3.49–6.10)	41 %
Beh. regulation	5.99 (4.87–7.12)	0.28 (0.11–0.44)	1.69 (0.65–2.88)	3.04 (1.43–4.64)	4.73 (3.43–6.03)	35 %
Opportunity						
Soc. Influence (IT)	4.51 (3.50–5.52)	0.22 (0.04–0.41)	1.01 (0.20–1.86)	3.71 (2.18–5.24)	4.73 (3.43–6.03)	21 %
Soc. Influence (SD)	2.94 (1.82–4.06)	0.25 (0.08–0.42)	0.74 (0.22–1.44)	3.87 (2.49–5.26)	4.62 (3.30–5.93)	16 %
Env. context/res	3.57 (2.52–4.62)	0.27 (0.09–0.45)	0.97 (0.34–1.73)	3.75 (2.33–5.17)	4.73 (3.43–6.03)	20 %
Motivation						
Beliefs Capabilities	1.74 (0.64–2.83)	0.28 (0.11–0.45)	0.49 (0.12–1.05)	4.26 (2.96–5.56)	4.75 (3.45–6.06)	10 %
Beliefs Consequences	1.43 (0.78–2.09)	0.24 (−0.04 – 0.53)	0.35 (−0.06 – 0.77)	4.31 (2.97–5.65)	4.66 (3.38–5.65)	7 % *
Goals	5.83 (4.71–6.95)	0.27 (0.10–0.44)	1.61 (0.62–2.68)	3.07 (1.46–4.69)	4.69 (3.37–6.00)	34 %
Intention	3.64 (2.65–4.63)	0.25 (0.06–0.45)	0.94 (0.23–1.81)	3.76 (2.30–5.23)	4.71 (3.40–6.01)	19 %

Coefficient = unstandardized Beta, * = not significant at p= 0.05, CI = 95 % Confidence Interval. Social influence was assessed from two perspectives; whether respondents perceived that they received support from the implementation team (IT) and whether they received support from the school district (SD).

### H3: Effect of the multifaceted implementation strategy on fidelity through capability, opportunity, and motivation

For the capability-related TDF domains, statistically significant direct effects were observed for all constructs. Moreover, statistically significant indirect effects were observed (knowledge: B = 0.81, CI 0.25 to 1.43; skills: B = 2.01, CI 0.73 to 3.37; and behavioral regulation: B = 1.69, CI 0.65 to 2.88). The direct effect remained significant after including the mediators, indicating that knowledge, skills, and behavioral regulation only partially mediated the effect of the multifaceted strategy on fidelity. The proportion-mediated statistics show that knowledge explained 17% of the total effect of the implementation strategy and fidelity, skills explained 41%, and behavioral regulation explained 35%.

For the opportunity-related TDF domains, statistically significant direct effects were observed for all domains. Moreover, statistically significant indirect effects for environmental context and resources (B = 0.97, CI 0.34 to 1.73), social influence from the implementation team (B = 1.01, CI 0.20 to 1.86), and social influence from the school district (B = 0.74, CI 0.22 to 1.44) were observed. The direct effect of the strategy on fidelity remained after the inclusion of the mediators, indicating that each of these domains partially mediated the effect of the implementation strategy on fidelity. 16% to 21% of the total effect of the implementation strategy’s effect on fidelity was mediated through the opportunity-related TDF domains.

For the motivation-related TDF domains, statistically significant direct effects were observed for all domains. Moreover, significant indirect effects were observed in beliefs about capabilities (B = 0.49, CI 0.12 to 1.05), intention (B = 0.94, CI 0.23 to 1.81), and goals (B = 1.61, CI 0.62 to 2.68). No significant indirect effect was observed for beliefs about consequences. Goals mediated 34% of the total effect of the implementation strategy on fidelity, 19% was mediated by intention, and 10% by beliefs about capabilities.

## Discussion

This study is one of the first to experimentally test the mechanisms of change of a multifaceted implementation strategy on fidelity to the Guideline for the Prevention of Mental Ill-Health at the Workplace. The findings support our hypothesis that the multifaceted strategy leads to higher guideline fidelity than the discrete strategy and that the effect of the multifaceted strategy on fidelity is partially mediated by capability, opportunity, and motivation as proposed by the COM-B model. The systematic theory-driven approach of testing the mechanisms of change by combining the overarching COM-B behavior change model with the corresponding TDF domains to identify underlying mechanisms makes this study an important contribution to advancing the field of both studying and understanding mechanisms of change. By showing how theories, models and frameworks can be integrated and applied, this study provides a roadmap for researchers seeking to explore the mechanisms of implementation strategies.

### The effect of the strategies on guideline fidelity

Our findings support hypothesis 1 showing that the multifaceted implementation strategy resulted in larger improvements over time in guideline fidelity, which were statistically different from those observed in the discrete strategy group. The results of this trial statistically confirm the trends observed in the previous trial favoring the multifaceted strategy [24]. Despite being relatively small, the improvements reflect the extent to which school staff observed the changes implemented by the school. The improvements observed in this more distal outcome of implementation success underscore the clinical meaningfulness of the improvements in guideline fidelity, as the observed changes were seen in the target group (school staff) who experienced a more systematic approach of the management of psychosocial risks in their work environment through the multifaceted strategy. Importantly, the observed improvements in fidelity suggest that these schools are also more likely to observe lower levels of psychosocial risks and improvements in staff health [24].

### The effect of the strategies on capability, opportunity, and motivation

Our findings align with hypothesis 2, showing that the multifaceted implementation strategy resulted in larger improvements in the TDF mediators corresponding to capability, opportunity, and motivation, which were statistically different from those observed in the discrete strategy group at T_3_. More pronounced improvements at T_3_ were observed for the capability-related TDF mediators. Smaller improvements were, however, observed for motivation-related TDF mediators at T_3_. Beliefs about the consequences, beliefs about capability, and intention, first decreased at T_2_ and slightly increased at T_3_. The findings suggest that a multifaceted implementation strategy containing educational strategies, implementation teams, improvement cycles, and implementation facilitation can improve TDF mediators corresponding to capability, opportunity, and motivation. By providing evidence of how a multifaceted strategy can improve and reinforce mediators over time, this study adds to the knowledge base on the causal pathways through which implementation strategies operate, which makes these findings an important contribution to the field [57, 58].

### Exploring the mechanism of change of the strategies

Our findings largely confirm hypothesis 3, showing that the effect of the strategy on fidelity was mediated through capability, opportunity, and motivation. Our findings align with the COM-B, which postulates that capability, opportunity, and motivation must be present for a behavior to occur [29]. The largest proportion of the strategy’s effect on fidelity was mediated through capability, specifically skills and behavioral regulation, and motivation, specifically goals, which mediated between 34–41% of the strategy’s effect on fidelity. This suggests that the strategy brought about change by activating the mechanisms of acquisition of skills to implement the guideline and fostering the ability to manage and control the behaviors necessary to implement the guideline. Moreover, the strategy activated the mechanism of enabling the prioritization of goals and action planning. Even though it seems likely that each implementation strategy activated multiple mechanisms, one possible explanation for the above findings could be that the activation of these mechanisms is a result of the PDSA strategy. The PDSA strategy probably enabled the implementation team to regulate and prioritize actions by providing a structure for planning, goal setting, monitoring, and feedback, which in turn enabled the teams to perform the necessary actions to achieve fidelity. This aligns with research suggesting that implementation strategies designed to address goal prioritization (such as iterative and evaluative strategies) can influence behavior by improving capability and motivation [59, 60]. This means that for schools to work in accordance with the guideline, school management and staff should have the necessary skills to do so and plan for action by setting goals, planning, feedback, and monitoring. Our findings are also in line with the barriers and enablers identified in the planning workshops conducted prior to the first trial, which informed the selection of the implementation strategies. During the planning workshop, difficulties in prioritizing and executing plans were identified as important barriers, and having a systematic approach was identified as a potential enabler. The results of the present trial indicate that the multifaceted strategy was indeed successful in addressing these identified determinants.

Our findings did not confirm hypothesis 3 with regards to the effect of the strategy on guideline fidelity being mediated by beliefs about the consequences. One possible explanation for this is the lack of difference in individuals’ beliefs about consequences between the multifaceted and discrete strategy groups. Both groups had high levels of belief about consequences directly after the educational meeting, which remained relatively high over time. This would suggest that the educational meeting resulted in an overall positive attitude towards the prevention of MHP through the guideline. Alternatively, this could also suggest that the strategy targeted a mechanism that was already activated [61]. School staff represent the occupational group with the second highest prevalence of MHP in Sweden and related sick-leave [62] and the need for schools to improve their work psychosocial work environment has been repeatedly highlighted [63]. The high scores in beliefs about consequences of working in accordance with the guideline to prevent MHP likely reflects the urgent need to change among schools.

### Implications for future implementation of the occupational guideline

This study adds to the lack of knowledge on effective implementation strategies to support the implementation of occupational guidelines to prevent MHP at the workplace [7, 8]. In summary, our results imply that training programs aimed at supporting relevant stakeholders (e.g., school managers and health and safety officers) with the systematic management of risk factors of MHP should not only focus on risk factors but also equip managers and stakeholders with the necessary skills and introduce tools for structured implementation. However, to enhance future implementation of occupational guidelines in this field, certain aspects of the multifaceted implementation strategy could be altered. First, alterations could be made to prevent the decrease in intention, beliefs about the consequences, and beliefs about capability, which occurred between T_1_ (educational meeting) and T_2_ (workshop 3). During this period, the implementation teams were instructed to conduct PDSA cycles to facilitate the implementation of the guideline within their school. Our unpublished observational data indicates that teams experienced difficulties in planning small changes and using the PDSA templates provided during the workshops. Instead of using the template as a planning tool, teams often planned their changes directly in their calendars. The decrease observed in beliefs about capability could reflect the teams’ perceived difficulties related to the PDSA cycles, which in turn influenced their beliefs about the consequences and their intention to implement the guideline. One aspect of the multifaceted strategy that, therefore, could be altered is to provide more support during the workshops and by the internal facilitator on how to plan for small changes and use the template to conduct the PDSA cycles. This suggested alteration corresponds well with our findings that skills, goals, and behavioral regulation mediated large proportions of the effect of the multifaceted strategy on guideline fidelity. As the PDSA strategy is one of the multifaceted strategies that address these TDF domains, future studies should explore how to improve the use of the PDSA strategy [43].

Secondly, alterations could be made to optimize and refine the multifaceted strategy by studying whether certain components of the strategy could be prioritized. This could be done by exploring whether all the discrete strategies included in the multifaceted strategy are needed and necessary in their current form to attain guideline fidelity. Future studies within the trial could map the components of each specific strategy to their primary target mechanism. For example, by mapping the educational meeting and workshops, which both contain components aimed at providing information on the guideline, to the target mechanism knowledge acquisition. If the mapping shows unnecessary overlap between the components and that only one of the components is needed to activate the mechanism, a choice could be made to remove one of these components [64]. This would increase the efficiency of the strategy and likely even the cost-effectiveness.

### Implication for other implementation efforts

Our study identified mechanisms of change by which a multifaceted implementation strategy can improve fidelity to an occupational guideline for the prevention of MHP at the workplace within a school setting. We do believe that our findings are generalizable to other settings and can have implications for other implementation efforts employing similar multifaceted strategies outside of the school setting. The generalizability of the strategy lies in the use of universal strategies (e.g., educational meeting and implementation team) [30] and that the strategy builds on the generic COM-B model, which assumes that for behavior change to occur, capability, opportunity, and motivation need to be present [29]. Due to the generic nature of the COM-B, this assumption holds for any behavior change irrespective of the intervention to be implemented and the setting in which it will be implemented [65]. The specific mechanisms that will be engaged in accomplishing the behavior change through the multifaceted strategy may, however, likely differ for different interventions and within different settings [65, 66]. For example, in the present study, large proportions of the strategy’s effect were mediated through skills, goals and behavioral regulation, which is in line with what was expected based on the pre-identified barriers and enablers. In other implementation efforts, there might be other mechanisms related to capability, opportunity and motivation that drive the behavior change. In this study, we demonstrate how to combine theories, models and frameworks to articulate and test mechanisms of change by specifying and testing contextually prioritized mediators, based on identified barriers and enablers, within broader and generalizable behavioral functions. This approach offers a replicable model for studies seeking to explain how and why strategies work.

It is worthwhile acknowledging that several contextual circumstances need to be taken into account for other implementation efforts. One of these contextual circumstances is the high need for evidence-based practice to prevent MHP among staff experienced by the schools, which was clearly reflected in the high overall reporting of beliefs about the consequences. Moreover, there are preconditions that need to be held for the employment of similar multifaceted strategies. The public-school setting in which the strategy was tested has provided certain structural preconditions that have been in place and enabled the execution of the multifaceted strategy. For example, the existence of a hierarchal infrastructure within the municipality containing individuals with clear roles and mandates, such as school directors and human resources (HR), has ensured an organizational climate for supporting schools with their implementation.

### Methodological considerations

The present study has several strengths and contributes on several fronts to the research agenda to advance the study of implementation mechanisms [18]. First, the study fulfills multiple requirements for identifying a mediator. In line with these requirements, the study applies 1) an experimental longitudinal design with an established timeline where changes in predictors precede changes in outcome, 2) random assignment to the groups, 3) use of a model/theory to select potential mediators, 4) assessment of multiple mediators, 5) measurement of mediators using validated instruments, and 6) use of a mediation model that estimates both direct and indirect effects [67]. A second strength is that a fully powered test of mechanisms was conducted. A third strength of our study is that it shows how an established, theory-driven measure can be applied to assess mechanisms of change. By testing the COM-B model’s proposed pathways to behavior change through its TDF domains and by using the DIBQ survey that is TDF mechanisms-specific, our study provides valuable knowledge on the measurement of mechanisms that can be generalized across multiple studies and illustrates the benefits of using theory to motivate and align mechanisms-related measurement choices.

There are also areas for improvement. First, the study is the first step in understanding the mechanism of change of this multifaceted strategy by testing pathways of change for each mediator separately. Although pathway analyses of separate mediators provide valuable knowledge regarding the contribution of each mediator and each mechanism of change, more studies are needed to understand how capability, opportunity, and motivation jointly contribute to improvements in fidelity to the guideline over time. Moreover, future studies should test the interrelationships between constructs to understand whether and how individual constructs, such as behavioral regulation and goals, interact to achieve implementation outcomes.

Next, in the development of the survey, those domains of the DIBQ questionnaire that were in line with our hypothesized mechanisms were included. Domains related to, for example, habitual behavior, such as the domain “nature of behavior,” which provides information on automatic processes, were not included. Research suggests that repetitive planning and enactment of plans can lead to automatic behavior [68, 69]; thus, additional change pathways not tested in this study could explain the effect of the strategy on fidelity. The same holds true for our choice to define the mechanisms of change through the COM-B model. Future studies should also look into other theories, such as organizational theories or combinations of theories, models, and frameworks that could have resulted in additional pathways [70]. Facilitation, for example, may work through pathways related to supportive organizational culture and climate [71] or through other mechanistic pathways [72]. The qualitative data that was collected during the trial will allow us to identify additional pathways of change that were not captured quantitatively [73], and to examine with more granularity how the implementation strategies in this study led to changes in capability, opportunity, and motivation [74, 75]. A third area to develop further relates to testing the effect and mechanisms of the discrete strategies included in the multifaceted strategy. Even though it was hypothesized in the study that the total bundle of strategies is necessary to result in improvements in guideline fidelity, it would be of interest to explore how each discrete strategy has contributed to improvements in fidelity. This is important, not the least from a cost and resource perspective, since the use of each additional strategy requires more resources. Although qualitative data within this trial will contribute valuable information on how the discrete strategies influence fidelity, we also encourage future quantitative studies that test how these strategies operate in other types of study designs to optimize their efficiency and effectiveness [76–79]. Finally, we acknowledge that further assessment of the fidelity measure’s validity is needed.

## Conclusion

The conclusion of this study is that capability, opportunity and motivation partially mediated the effect of the multifaceted implementation strategy on fidelity via their matching TDF domains, which is in line with the anticipated pathways of the COM-B behavior change model. The study responds to calls for experimental evidence on how a multifaceted strategy operates to achieve implementation outcomes. Qualitative studies are needed to provide knowledge on the mechanisms of discrete implementation strategies and how they jointly function to improve fidelity. This study provides valuable knowledge on how managers can best be supported to work in accordance with occupational guidelines to prevent MHP, by demonstrating the effectiveness of a multifaceted approach relative to one-time education, which is the most common support provided to managers to acquire knowledge of how to manage the psychosocial work environment.

## Supplementary Information

Supplementary Material 1.

## Data Availability

Not applicable.

## Abbreviations

DIBQ: Determinants of Implementation Behavior Questionnaire
EBP: Evidence-Based Practice
ERIC: Expert Recommendations for Implementing Change
LMM: Linear Mixed Modeling
MHP: Mental Health Problems
PDSA: Plan-Do-Study-Act
TDF: Theoretical Domains Framework
